# Grand Challenges in Understanding Gut Microbes

**DOI:** 10.3389/fmicb.2021.752829

**Published:** 2021-10-05

**Authors:** Knut Rudi, Liping Zhao

**Affiliations:** ^1^Faculty of Chemistry Biotechnology and Food Sciences, Norwegian University of Life Sciences, Ås, Norway; ^2^Rutgers, The State University of New Jersey, New Brunswick, NJ, United States

**Keywords:** digestion and gut health, gut microbiota (GM), sequencing, host microbe interactions, coevolution

The importance of digestion and gut health has been recognized since ancient times through ideas coined by Aristotle, the father of modern medicine. The role of gut microbes in digestion, however, was not truly unveiled until the use of germfree animals in the middle of the twentieth century (Reyniers et al., 1946). Introduction of single microbes, or defined assemblages of microbes to germfree hosts, enabled the first detailed studies on how gut microbes can be related to health and disease (Reyniers and Sacksteder, 1958). Early breakthrough discoveries showed that microbes can harvest energy from compounds for which the host lacks the enzymatic machinery needed to degrade them, with microbiota derived short chain fatty acids being an important energy source for many animals (Bergman, 1990). It has also been shown that the gut microbiota can induce their own nutrient production from the host epithelial cells (Bry et al., 1996). Taken together, these early discoveries highlight the mutualistic relation between gut microorganisms and the host.

Through the introduction of new culturing and new analytical approaches, in particular second and third generation sequencing, we have made major breakthrough advancements during the twenty-first century in the understanding of the role of microbes in our gut (Rodríguez et al., 2015). The most compelling discoveries perhaps relate to the findings that gut microbes can affect our mood and behavior (Bravo et al., 2011; Naseribafrouei et al., 2014), are an essential component of immunological imprinting during infancy (Olin et al., 2018), and influence non-communicable diseases (Zhao et al., 2018).

Although new technologies have enabled deeper and more detailed insight into aspects of host-microbe associations, these associations mainly represent snapshots in time and space. Fundamental knowledge such as generation time of microbes and flux of metabolites across the gut remains largely unknown. We also still lack fundamental knowledge about the ecology of the gut associated bacteria and the interplay with host. Finally, we have limited knowledge related to the mobile genetic elements in the gut microbiota. Determining such parameters will be crucial for mechanistic understanding of microbes in our gut, and ultimately in deriving causal mechanistic models.

In this grand challenge description, we will highlight some of these fields where we believe we still have major knowledge gaps that need to be filled in the future.

## Host Microbe Interactions

Perhaps the most well-established host association of the gut microbiota relates to the dependence of colonocytes on butyrate produced by the gut microbiota. In addition to being an important energy source, butyrate has a major role in immune modulation (Furusawa et al., 2013). A particularly important aspect is the timing of the establishment of butyrate producing gut microbiota during infancy (Nilsen et al., 2020). An aspect that needs further attention in the future is the interplay between metabolite production and developmental processes (Cait et al., 2019; Jena et al., 2020).

Recent theories suggest that we should treat the host and the microbiome as a single unit (Postler and Ghosh, 2017). However, there seems to be a weak link between gut microbiota composition and host genetics (Dvergedal et al., 2020, Rothschild et al., 2018), implying that host genetic factors may not play a major role in shaping the gut microbiota composition. A major contributor to lack of heritability is epigenetic factors (Sharma et al., 2020). Thus, we believe epigenetic associations will be an important field for future gut microbiota research. The genetic and epigenetic association with the gut microbiota, however, is a field still in development (Sandoval-Motta et al., 2017), with several likely breakthrough discoveries in the future.

## Host Microbe Coevolution

Since the emergence of vertebrates, there has been an intimate coevolution between the vertebrate host and the microorganisms living in the gut, with microorganisms providing essential functions to the host. A particularly compelling question is to what extent gut microbially provided functions can contribute to vertebrate speciation (Moeller and Sanders, 2020). We therefore believe further investigations should be focused on unraveling detailed coevolutionary patterns between the host and gut associated microbes. We also believe identifying mechanisms for coevolution will be of key importance in the future.

## Understanding Flux Rates Of Gut Microbiota Derived Metabolites

A main knowledge gap relates to the flux rates of microbiota derived metabolites in the gut, in particular related to short chain fatty acids (SCFAs). Since most of the SCFAs are immediately absorbed, and also partly metabolized by the gut, the true production rate is very difficult to estimate (Bergman, 1990). Despite limited knowledge about fluxes, SCFAs have been related to a range of important health states (Parada Venegas et al., 2019; He et al., 2020). With future development of metabolic tracers and *in vivo* measurements, we foresee that detailed flux measurements will aid in increasing our mechanistic knowledge in understanding the role of gut microbiota derived metabolites in health and disease.

## Ecological Principles In The Gut

Gut microbiome is not the “-ome of all microbial genes” but the “biome of all microbes” living in the human gut. As a microbial ecosystem, microbiome is a complex adaptive system in which the most basic building blocks organize themselves into a higher-level structure that may work together to contribute to community level emergent functions relevant to host health. We need more fundamental knowledge about the ecological principles in host-microbe interactions in the gut. Still, the question of whether the gut microbiota is mainly controlled by bacterial-bacterial interactions or by top-down interactions by the host remains unresolved (Ley et al., 2006). In particular, the extent of positive selection in the gut, as exemplified by the human milk oligosaccharides and sugar induction from gut epithelial cells, needs more attention (Avershina and Rudi, 2013). We also need more knowledge on transmission and assemblage of the gut microbiota at host population level. This knowledge is particularly important with respect to fragmentation of ecosystems and in the implementation of hygiene barriers. In human and other populations we risk eradicating important microbes that serve essential functions in the gut (Wibowo et al., 2021). Furthermore, we need to understand causality of the ecological interactions for the microorganisms in the gut (Wu et al., 2021). We believe future research should be targeted toward how we can integrate our lifestyle with the ecological processes of the gut.

## Reservoir For Antibiotic Resistance

If no actions are taken, antibiotic resistant bacteria are estimated to kill more people than cancer in 2050 (de Kraker et al., 2016). The commensal gut microbiota of human and animals represent a major reservoir of antibiotic resistant microbes (Anthony et al., 2020). In particular, the use of antibiotics as growth promoters in food producing animals has been a major cause of spread of antibiotic resistance (Aarestrup, 2012). Despite the fact that antibiotic usage for animal growth promotion has been banned in several countries for many years, antibiotic resistance is still persistent. A possible explanation could be the presence of selfish mobile genetic elements within the gut microbiota (Ravi et al., 2015). These elements can transmit resistance, even in the absence of antibiotic selection (Hagbo et al., 2019). A field that deserves future investigation is the role of the commensal gut microbiota mobilome in the persistence and spread of antibiotic resistance.

## Culturing The Unculturable

Culturing of microorganisms is essential to determine mechanistical associations and causality in the gut (Zhao and Zhao, 2021). For the human gut microbiota, there has been major advances in culturing approaches. In particular, culturing techniques have opened the possibility to analyze the strictly anaerobic microbes (Lewis et al., 2020).

A particularly fruitful approach has been to use culture independent approaches to identify key properties that will enable culturing (Nayfach et al., 2019). For the human microbiome we are therefore at a stage where we have developed a comprehensive understanding of most of the genomes in the human gut (Hiseni et al., 2021). In the future, we think that the lessons learned for culturing of human gut microbes should also be expanded to other vertebrates in order to develop a comprehensive understanding of the microbes living in the vertebrate gut.

## Mechanistic Models

The ultimate goal will be to build mechanistic models for how the gut microbiota interact with the host. For the human gut, the first genome scale metabolic models have already been developed (Magnúsdóttir et al., 2016), with the development of a comprehensive database in relation to nutrition and disease (Noronha et al., 2019). Based on this information, mechanistic ecological models have also been derived in order to explain the colonization of the human infant gut (Angell and Rudi, 2020). There are also recent developments in spatio-temporal models of the gut microbiota (Dukovski et al., 2020), which we foresee will be of major importance in the future for a true mechanistic understanding of the vertebrate gut microbiota.

## Standardization Of Quality Control Measures For Microbiome Studies

Huge amounts of microbiome data are being generated from large scale microbiome projects as well as from hundreds of research groups who are moving into the microbiome field. It becomes urgent to ensure that all the microbiome datasets are generated according to the same quality control (QC) standards so that they can be compared and integrated, which can lead to novel global insights and findings. A few projects have been conducted to develop standardized protocols for microbiome analysis. However, many questions and challenges remain. For sample collection, most projects use stool collection kits that rely on a fixation buffer to inactivate microbes and prevent them from further growth. However, it is very rare for both kit developers and their users to test the kits with microbiological methods to ensure that no microbial cells will survive/grow inside the fixation buffer during the handling time at room temperature. The field needs to establish a QC protocol that combines a plate count method to assess cell survival/growth in the kit and DNA/RNA sequencing to assess changes of microbiota composition and gene expression pattern for evaluation and selection of stool collection kits for microbiome projects. For nucleic acid extraction, each stool sample has a different ratio of Gram-positive cells that are difficult to lyse. Current cell lysing protocols for microbiome analysis usually apply the same protocol to all stool samples without providing vigorous QC tests to determine whether all types of cells in different stools can be lysed to ensure a high and consistent DNA/RNA recovery rate. Such QC data is needed before a DNA/RNA extraction protocol is adopted for a microbiome project. For large scale microbiome projects in which samples need to be sequenced in many different runs, the same set of reference stool samples should be repeatedly sequenced in each sequencing batch. A set of stool samples that show both individual variations and responses to relevant interventions in their microbiome datasets can be selected as the reference stool samples to detect, assess and remove batch effect if they are sequenced in each and every sequencing run for the same project and ideally also for different projects. Microbiome projects should be designed and conducted as discovery science projects. If microbiome analyses rely exclusively on databases to assign taxa or gene functions, the unclassified or unannotated part of the dataset will be excluded from the following analysis, which limits the findings to what is already known. To make true discoveries, the field should develop reference-free and database-independent analytical methods for microbiome datasets.

## Concluding Remark

Vertebrate microbiota represents a burgeoning field which not only may provide new answers to many basic questions such as co-evolution as a fundamental mechanism for speciation, but also may bring new insights on managing the health of Humans and our Earth Planet. The topics highlighted here do not represent a comprehensive list of potential research targets for the future. In particular, we believe the field will diversify with respect to new host organisms outside those commonly investigated. We believe the future will not just belong to the Centers for Big Microbiome Science, but also individual labs with targeted niches within the field of the vertebrate gut microbiota. For individual labs to make an impact in this data-driven field, we would like to encourage the development and adoption of minimum quality control standards for study design, data collection and analysis in this Section. Such a new methodological framework will ensure that data generated from Big Centers and small labs on various vertebrate microbiota can all be integrated for generating a new global view on microbiome and health.

## Author Contributions

All authors listed have made a substantial, direct and intellectual contribution to the work, and approved it for publication.

## Funding

This work was partly financed by the Research Council of Norway through Project No. 301364, UnveilMe: Unveiling the Role of Microbial Metabolites in Human Infant Development.

## Conflict of Interest

The authors declare that the research was conducted in the absence of any commercial or financial relationships that could be construed as a potential conflict of interest.

## Publisher's Note

All claims expressed in this article are solely those of the authors and do not necessarily represent those of their affiliated organizations, or those of the publisher, the editors and the reviewers. Any product that may be evaluated in this article, or claim that may be made by its manufacturer, is not guaranteed or endorsed by the publisher.
